# Reticulate hyperpigmentation in systemic sclerosis: a case report and review of the literature

**DOI:** 10.1186/s13256-015-0697-2

**Published:** 2015-09-28

**Authors:** Mati Chuamanochan, Andrea L. Haws, Penvadee Pattanaprichakul

**Affiliations:** 1 Department of Dermatology, Faculty of Medicine Siriraj Hospital, Mahidol University, 2 Prannok Rd, Bangkoknoi, Bangkok 10700 Thailand; 2 Department of Dermatology, University of Texas Health Science Center, 6655 Travis St., Suite 980, Houston, TX 77030 USA

**Keywords:** Systemic sclerosis, Scleroderma, Reticulate hyperpigmentation

## Abstract

**Introduction:**

Systemic sclerosis is a systemic connective tissue disease with variable cutaneous presentations. Although pigmentary disturbances have been described in systemic sclerosis, a reticulate hyperpigmentation has only been reported in one case of systemic sclerosis to date.

**Case presentation:**

We describe a previously healthy 51-year-old Thai woman who presented with a reticulate hyperpigmentation affecting her trunk and extremities, together with sclerodactyly and proximal sclerosis, resulting in a new diagnosis of systemic sclerosis.

**Conclusions:**

To date, the pathogenesis of reticulate hyperpigmentation in systemic sclerosis remains unclear. Increased melanin synthesis and altered thermoregulatory mechanism are proposed to be involved in the pathogenesis of this presentation. This case represents an unusual cutaneous feature of reticulate hyperpigmentation in the setting of systemic sclerosis.

## Introduction

Systemic sclerosis (SSc) is a multisystem rheumatic disease with a variable clinical presentation. Clinical diagnosis is mainly based on the presence of skin thickening and variable involvement of internal organs. Various cutaneous pigmentary alterations have been described in SSc [1], including a diffuse, generalized hyperpigmentation with accentuation in sun-exposed areas, a vitiligo-like depigmentation with perifollicular hyperpigmentation, and a combined hyper- and hypopigmentation in the areas of sclerosis [2–6]. The pattern of reticulate hyperpigmentation in SSc has been rarely reported in the literature, and has an unclear pathogenesis. We report an unrecognized pigmentary abnormality in a 51-year-old patient with SSc who presented with a reticulate hyperpigmentation affecting the trunk and all extremities. To date, there have been only a few cases of reticulate hyperpigmented scleroderma reported in the English literature.

## Case presentation

**Fig. 1:**
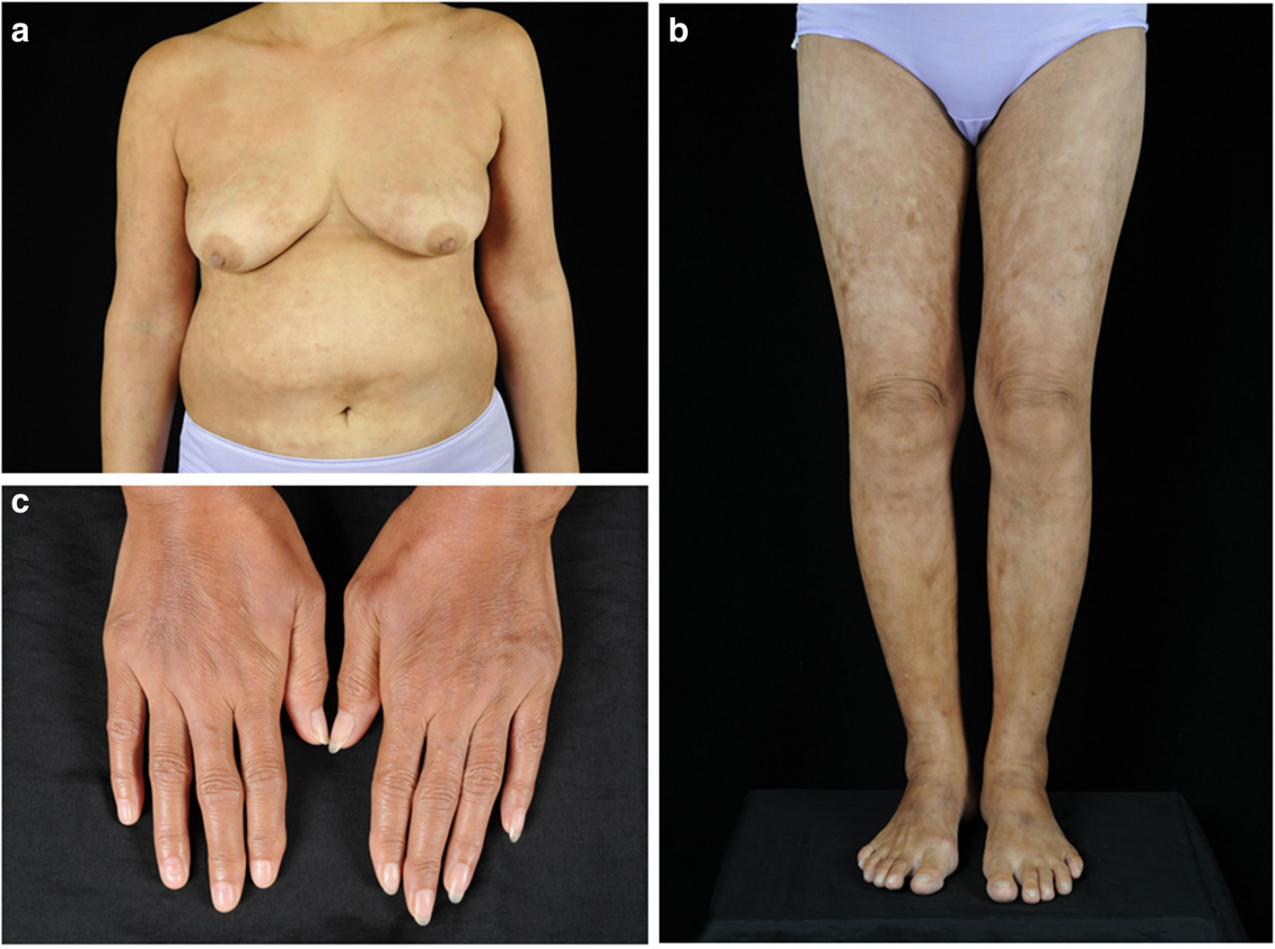
Clinical presentation. **a**, **b** Widespread reticulate hyperpigmentation over the trunk and extremities with discrete erythematous indurated plaques over the chest wall and anterior abdomen, **c** Sclerodactyly with cool periphery of all fingers

**Fig. 2:**
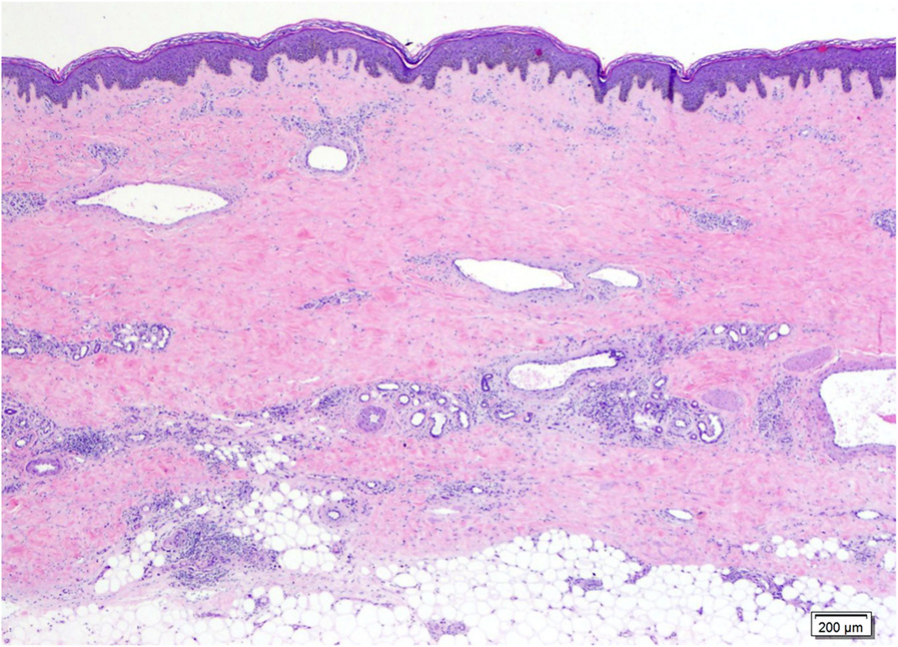
Histopathological findings. A biopsy from the hyperpigmented induration on the right thigh showed increased basal pigmentation with pan-dermal sclerosis. Mild superficial and deep perivascular and perieccrine lymphocytic infiltrate was observed with some dilated dermal blood vessels and decreased adventitious fat surrounding sweat glands. (Hematoxylin and eosin, ×10 objective)

Our patient, a 51-year-old Thai woman, presented with a 1-year history of progressively evolving skin pigmentation over her body, which had become accentuated over her trunk and extremities during the past few months. She also complained of appetite loss resulting in an unintentional weight loss of 8kg (17.6lbs) in 1 year, joint pain in her wrists and ankles, and bilateral hand swelling. Our patient also reported the presence of Raynaud’s phenomenon (RP) during the wintertime that had occurred about 5 years prior to the skin change of the hands. There were no other remarkable systemic symptoms. Our patient had been previously healthy and did not take any medications. There was no familial history of a similar skin condition or history of chemical exposure in this case. A physical examination revealed a generalized, reticulate hyperpigmentation and indurated erythematous plaques over her trunk and extremities. Additionally, there was sclerosis of her proximal fingers, sclerodactyly with pitted scars on some fingertips, and Raynaud’s phenomenon (Fig. 1a-c). Nailfold capillaroscopy was performed and showed few capillary loops dilatation without significant tortuosity. Telangiectasia and calcinosis cutis were absent in this patient. There was no evidence of additional systemic involvement. Laboratory investigations revealed normal white blood cell count, creatinine, electrolytes, fasting blood sugar, thyroid function, and liver function. An elevated erythrocyte sedimentation rate (ESR) of 40mm/hour (reference range 0–20mm/hour), a positive antinuclear antibody (ANA) titer of 1:640 (coarse-speckled pattern), and a positive rheumatoid factor (RF) >130IU/mL (reference range; <12.5IU/mL = negative, 12.5–20.5IU/mL = borderline, >20.5IU/mL = positive) were detected. Anti-centromere antibody (ACA), anti-topoisomerase I antibody (anti-Scl-70 Ab), anti-dsDNA antibody, anti-RNP antibody, anti-cardiolipin antibody (immunoglobulin G (IgG) and IgM), lupus anticoagulant, anti-β_2_-glycoprotein 1 antibody (anti-β2-GP1 Ab), and anti-cyclic citrullinated peptide antibody (anti-CCP Ab) tests revealed negative results. Urinalysis, chest X-ray, pulmonary function test, and upper gastrointestinal endoscopy results were normal. A skin biopsy specimen from her right calf corresponding to the hyperpigmented induration showed hyperpigmentation of basal keratinocytes and broad sclerotic collagen bundles involving full thickness of the dermis, replacing adventitious fat, and extending into the subcutis with mild septal thickening (Fig. 2). Some ectatic capillary blood vessels were found in the superficial and deep reticular dermis without evidence of vasculitis or vasculopathy. A superficial and deep perivascular infiltrate composed of lymphocytes, plasma cells, and some eosinophils was noted. Based upon the clinical combination of sclerodactyly with sclerosis of the proximal fingers, generalized reticulate hyperpigmentation, and histopathological finding of pan-dermal sclerosis, reticulate hyperpigmented systemic sclerosis was diagnosed.

## Discussion

Reticulate hyperpigmentation is characterized by mottled patterns of cutaneous hyperpigmentation. The etiology is varied from congenital to acquired conditions. An initial approach to identify the causes of reticulate hyperpigmentation depends on the characteristic onset of the disease, the distribution of lesion, and the associated clinical findings. Further investigations including a skin biopsy may be helpful for the definitive diagnosis [1]. There are several patterns of pigmentary alteration previously described in sclerodermic patients, which are: i) diffuse generalized hyperpigmentation, similar to Addison’s disease; ii) focal depigmentation with perifollicular hyperpigmentation, resembling vitiligo; iii) localized hypo- and hyperpigmentation in localized sclerotic skin; iv) streaky hyperpigmentation over blood vessels on a background of depigmentation on the legs and temporal scalp; and v) the most recent condition termed “reticulate hyperpigmented scleroderma” [2–6]. The pathogenesis of these hyperpigmentation abnormalities remains unclear, but some proposed hypotheses include increased keratinocyte-derived endothelin-1 (ET-1), increased melanin synthesis; increased secretion of melanocytic growth factors by fibroblasts and endothelial cells; and a thermoregulatory mechanism which results in a hyperpigmentation over vessels (“streaky hyperpigmentation”) on a background of depigmented patches [4, 7–9]. Histopathological findings to explain hyperpigmentation in SSc have been previously reported as increased epidermal melanin and pigmentary incontinence with an increased number of dermal melanophages in the superficial dermis [10]. However, in this present case, the lesional skin biopsy showed prominent basal hyperpigmentation without evidence of melanin incontinence or increased dermal melanophages.

The presence of some indurated erythematous plaques over the trunk and extremities is quite uncommon regarding sclerotic skin change in SSc, and therefore the differential diagnosis with generalized morphea or other scleroderma-mimic conditions such as eosinophilic fasciitis, sclerodermiform genodermatoses, scleroderma-like syndromes induced by environmental factors, scleroderma diabeticorum, nephrogenic systemic fibrosis, graft-versus-host disease, and scleroderma-like lesions in malignancies also is concerned. Thus, the absent history of underlying systemic diseases including diabetes, chronic kidney disease, malignancy or substance exposures and the recent onset of cutaneous symptoms would render the diagnosis of SSc regardless of other etiology. Moreover, the presence of sclerodactyly, RP and abnormal nailfold capillaroscopy support the diagnosis of SSc in our case.

To date, there have been only three reported cases of reticulate hyperpigmented scleroderma. Of these reports, two cases were the result of melphalan-induced localized reticulate scleroderma, which occurred secondary to isolated limb perfusion for treatment of malignancies in patients who did not have a history of SSc or other connective tissue diseases [2, 3]. These patients presented with localized porcelain-white sclerotic bands in a fishnet pattern, which was due to melphalan-induced endothelial injury. Ee *et al.* reported the only other known case to date of reticulate hyperpigmented scleroderma arising in the setting of SSc, and was that of a 48-year-old woman [2]. The pathogenesis remains unclear and further study is required to identify the mechanism of reticulate pigmentary change in SSc. The thermovascular influence, as suggested by Jawitz *et al*., is one of the possible mechanisms [4]; however, the skin biopsy in our patient did not reveal the presence of vasculitis or vasculopathy. The only vascular-related changes seen in the biopsy was the dilation of dermal vasculature, which was surrounded by sclerotic collagen within the dermis.

In summary, our patient is the second reported case of reticulate hyperpigmented scleroderma, and the first case to provide outcome information. Our patient has been treated and responded well to a combination therapy of topical potent corticosteroids, ultraviolet A1 (UVA1) phototherapy, and daily oral medication with colchicine 1.2mg, aspirin 80mg, nifedipine 30mg, vitamin E 400mg, and hydroxychloroquin 200mg. Improvement of the skin hardening and RP were observed after 3 months of treatment. However, our patient reported occasional exacerbation of RP during the winter. The comparative case information between the previous case reports and our case is shown in Table 1.

**Table 1:** Clinicopathological presentation and management of reticulate hyperpigmented scleroderma

	Previous case [2]	Previous case [3]	Previous case [3]	Present case
Age (year) , sex	48, female	47, male	43, male	51, female
Signs and symptoms	Raynaud’s phenomenon, dsyphagia, sclerodactyly, periungual telangiectasias, mask-like facies with perioral radial furrows; new diagnosis of systemic sclerosis	Porcelain-white, sclerotic bands in a reticulate pattern and painful ulcerations; associated with melphalan	White reticular sclerotic bands with painful ulcerations; associated with melphalan	Raynaud’s phenomenon, sclerodactyly
Area of scleroderma involved	Trunk, thighs, upper and lower limbs	Left thigh and upper calf	Right thigh	Proximal sclerosis involving trunk and extremities
Area of reticulate hyperpigmentation	Chest, abdomen and back	Medial aspect of the left thigh and upper calf	Medio-popliteal aspect of the right thigh	Trunk and extremities
ANA	Negative	n/a	n/a	Positive, titer 1:640 (speckled pattern)
Anti-centromere Ab	Negative	n/a	n/a	Negative
Anti-Scl-70 Ab	Negative	n/a	n/a	Negative
Anti-cardiolipin Ab	Negative	n/a	n/a	Negative
Histological findings				
Epidermis	Epidermal atrophy and basal pigmentation	Epidermal atrophy	n/a	Basal hyperpigmentation
Dermis and subcutis	Thickened sclerotic collagen, marked pigmentary incontinence with numerous melanophages in the upper dermis, septal thickening	Thickened, intensely eosinophilic and closely packed collagen bundles	n/a	Broad sclerotic collagen bundles in dermis replacing adventitious fat, superficial and deep perivascular lymphoplasmacytic infiltrate with few eosinophils, mild septal thickening
Treatment	n/a	Topical corticosteroids and antibiotics under a hydrocolloid dressing	Hydrocolloid dressing and topical antibiotics	Topical corticosteroid, colchicine, aspirin, nifedipine, vitamin E, hydroxychloroquin UVA1 phototherapy
Follow-up	n/a	Recovery of the ulcers	n/a	Improvement of thickened skin and Raynaud’ s phenomenon

*ANA* antinuclear antibody, *n/a* not available, *Ab* antibody, *anti-Scl-70 Ab* anti-topoisomerase I antibody, *UVA1* ultraviolet A1

## Conclusions

Reticulate hyperpigmented scleroderma is a distinctly rare cutaneous presentation in association with systemic sclerosis. The pathogenesis is uncertain. Unlike the previously reported case of reticulate hyperpigmentation in association with systemic sclerosis, our patient failed to demonstrate the presence of vascular alterations or melanin incontinence in the skin biopsy. Further studies are necessary to evaluate the mechanism of this peculiar pigmentary change in systemic sclerosis.

## Consent

Written informed consent was obtained from the patient for publication of this case report and any accompanying images. A copy of the written consent is available for review by the Editor-in-Chief of this journal.
